# Semi-Supervised Support Vector Machine for Digital Twins Based Brain Image Fusion

**DOI:** 10.3389/fnins.2021.705323

**Published:** 2021-07-09

**Authors:** Zhibo Wan, Youqiang Dong, Zengchen Yu, Haibin Lv, Zhihan Lv

**Affiliations:** ^1^College of Computer Science and Technology, Qingdao University, Qingdao, China; ^2^R&D Department, Qingdao Haily Measuring Technologies Co., Ltd., Qingdao, China; ^3^North China Sea Offshore Engineering Survey Institute, Ministry Of Natural Resources North Sea Bureau, Qingdao, China

**Keywords:** semi-supervised support vector machines, brain image, digital twins, image segmentation, improved AlexNet

## Abstract

The purpose is to explore the feature recognition, diagnosis, and forecasting performances of Semi-Supervised Support Vector Machines (S3VMs) for brain image fusion Digital Twins (DTs). Both unlabeled and labeled data are used regarding many unlabeled data in brain images, and semi supervised support vector machine (SVM) is proposed. Meantime, the AlexNet model is improved, and the brain images in real space are mapped to virtual space by using digital twins. Moreover, a diagnosis and prediction model of brain image fusion digital twins based on semi supervised SVM and improved AlexNet is constructed. Magnetic Resonance Imaging (MRI) data from the Brain Tumor Department of a Hospital are collected to test the performance of the constructed model through simulation experiments. Some state-of-art models are included for performance comparison: Long Short-Term Memory (LSTM), Convolutional Neural Network (CNN), Recurrent Neural Network (RNN), AlexNet, and Multi-Layer Perceptron (MLP). Results demonstrate that the proposed model can provide a feature recognition and extraction accuracy of 92.52%, at least an improvement of 2.76% compared to other models. Its training lasts for about 100 s, and the test takes about 0.68 s. The Root Mean Square Error (RMSE) and Mean Absolute Error (MAE) of the proposed model are 4.91 and 5.59%, respectively. Regarding the assessment indicators of brain image segmentation and fusion, the proposed model can provide a 79.55% Jaccard coefficient, a 90.43% Positive Predictive Value (PPV), a 73.09% Sensitivity, and a 75.58% Dice Similarity Coefficient (DSC), remarkably better than other models. Acceleration efficiency analysis suggests that the improved AlexNet model is suitable for processing massive brain image data with a higher speedup indicator. To sum up, the constructed model can provide high accuracy, good acceleration efficiency, and excellent segmentation and recognition performances while ensuring low errors, which can provide an experimental basis for brain image feature recognition and digital diagnosis.

## Introduction

Now that science and technology advances quickly, Artificial Intelligence (AI), Big Data (BD), the Internet of Things (IoT), and Deep Learning (DL) have been accepted in all walks of life. Health is vital to the happy life of people. Medical intelligentization has improved health monitoring techniques notably. Brain tumors have severely harmed people’s health.

Statistics suggest that the annual mortality rate of brain cancer in China is second only to gastric cancer and lung cancer. About 80,000 people die from brain cancer each year, accounting for about 22.5% of the world’s total number of brain cancers (Zhou et al., 2019; Kang et al., 2021). In only a few decades of the 21st century, the number of people diagnosed with brain cancer continues to increase, especially in Asian countries (Zheng et al., 2020). Thus, brain health has become the focus of brain disease experts, researchers, and brain surgeons.

Medical imaging techniques play a vital role in clinical diagnosis with the successive introduction of medical imaging equipment and the continuous development of computer science and technology. Results of brain disease diagnosis depend on brain image acquisition and accurate interpretation. However, due to the large number of patient groups, the number of brain and other medical images keeps increasing dramatically (Tobon Vasquez et al., 2020). Manual image reading is apparently inefficient, and the workload for clinicians is unbearable. Therefore, using computer-aided diagnosis to extract features from medical images is of great significance in improving diagnostic accuracy and reducing clinicians’ workload.

Image segmentation is one of the necessary steps of brain image fusion, so it is necessary to segment and extract the features of the image. There are many approaches to extract brain image features; currently trendy ones include Region Growing (RG), Level Set, Fuzzy Clustering, and Machine Learning (ML) (Aheleroff et al., 2020; Angin et al., 2020; Li et al., 2020; Pan et al., 2021). However, these approaches require manual image segmentation, consuming lots of human resources and materials. As a burgeoning AI algorithm, ML extracts data features and investigates data relationships through self-learning on brain images, which dramatically increases image segmentation accuracy and stability while improving efficiency. As Digital Twins (DTs) evolve, their applications in brain image segmentation have enhanced data generation speed and scale, leading to a remarkable increase in big data processing complexity and brain image analysis (Gudigar et al., 2019). Digital twins can map complex brain images from real space to virtual space, and it is of great significance to extract features, segment, and fuse images.

To sum up, AI applications in medical fields, such as brain image processing, can help clinicians choose surgical treatment or radiotherapy and chemotherapy treatment options for patients while avoiding the steps of direct surgical resection of all tumors, which greatly reduces the risk of treatment. The innovative points are (1) the Semi-Supervised Support Vector Machines (S3VMs) proposed regarding the massive volume of unlabeled brain image data, (2) the improved AlexNet, and (3) the brain image fusion DTs diagnosis and forecasting model constructed based on S3VMs and improved AlexNet, in an effort to provide theoretical support for brain image feature recognition and digital diagnosis.

## Recent Works

### ML Applications

Now that complex data grow explosively, big data mining and analysis functions, especially DL, are demanded increasingly, which has been explored by many scholars. Yan et al. (2018) put forward Device ElectroCardioGram (DECG) and constructed a forecasting algorithm for the remaining service life of industrial equipment based on Deep Denoising Autoencoder (DDA) and regression calculation. Comparison with traditional factory information models validated the feasibility and effectiveness of the proposed algorithm (Yan et al., 2018). Wu et al. (2019) adopted deep learning algorithms in medical imaging to understand patients’ physical condition, thereby improving the disease treatment. Ghosh et al. (2019) developed a DL approach for molecular excitation spectroscopy forecasting. They trained and evaluated Multi-Layer Perceptron (MLP), Convolutional Neural Network (CNN), and Deep Tensor Neural Network (DTNN) to analyze the electronic state density of organic molecules. Eventually, they discovered that real-time spectroscopic forecasting could be performed on the structure of tiny organic molecules, and potential application molecules could be determined (Ghosh et al., 2019). Shao and Chou (2020) developed a CNN-based forecasting factor for the subcellular localization of viral proteins, called Ploc-Deep-mVirus, for the Coronavirus Disease 2019 (COVID-19) currently spreading globally. They found that this forecasting factor was particularly suitable for processing multi-site systems, and its forecasting performance was remarkably better than other advanced forecasting indicators (Shao and Chou, 2020). Ahmed et al. (2020) applied DL approaches to the clinical or behavioral recognition of Autism Spectrum Disorder (ASD). They designed an image generator, which could produce a single-volume brain image from the entire brain image by separately considering the voxel time point of each subject. Eventually, performances of four different DL approaches, as well as their corresponding integrated classifiers and the algorithms, were assessed. Results demonstrated a better effect on a large-scale multi-site brain imaging dataset (bedict) (Ahmed et al., 2020).

### Research Progress of Image Fusion DTs

Qi and Tao (2018) applied DTs to image information physical integration of the manufacturing industry. They compared the similarities and differences between BD and DTs from the overall and data perspectives. Consequently, they discussed using DTs to promote smart manufacturing (Qi and Tao, 2018). As embedded sensors, BD processing, and cloud computing got advanced, Barricelli et al. (2019) applied DTs to medical image diagnosis and recognition, and consequently, validated its effectiveness and feasibility. Liu et al. (2019) put forward a Cloud DTs Healthcare (CloudDTH) architecture to monitor, diagnose, and forecast image data collected by wearable medical devices to forecast individual health status, thereby achieving individual health management, especially the health management of seniors. Li et al. (2021) proposed a novel semantic-enhanced DTs system for accurate and fine-grained real-time monitoring of Robot-Environment Interaction (REI). This system adopted the robot’s external perception and ontology perception as the mechanism of interaction state monitoring and semantic reasoning. Meanwhile, they proposed a multi-feature fusion visual relationship detector and introduced a lightweight spatial relationship dataset to measure the visual relationship between entity pairs. They evaluated the real-time monitoring ability and semantic reasoning ability of the system and verified the effectiveness and feasibility of the scheme (Li et al., 2021).

The above works reveal that most ML algorithms applied in the medical field collect image data from historical cases. In contrast, DTs are mappings from virtual space to real cases. There are very few combinations of DTs and ML in the medical field. Hence, ML approaches and DTs are introduced for brain image fusion in the present work, which is of great significance to the accurate identification and diagnosis of brain images.

### ML-Based Brain Image Fusion DTs Diagnosis and Forecasting

At present, although many achievements have been made in the fusion of medical images such as brain tumors, the results are still unsatisfactory, and the application effect in clinical medicine is extremely limited. As one of the effective methods of brain image fusion, image segmentation technology has great practical value and research significance in the diagnosis, prediction, and feature extraction of brain tumor and other medical images.

### Demand Analysis of Brain Image Fusion DTs Diagnosis and Forecasting

With the aging of the population, the number of patients is increasing, and the number of medical images is growing. It has become a new direction to diagnose and treat diseases by using computer technology such as digital twins to assist the recognition and prediction of brain images. Sun et al. (2020) at present, common medical imaging techniques include X-ray, Computed Tomography (CT), Magnetic Resonance Imaging (MRI), Ultrasound Imaging (UI), Positron Emission Tomography (PET), and Molecular Imaging (MI) (Wang et al., 2020). MRI is very suitable for brain tumor research and diagnosis because of its non-invasiveness, high soft-tissue contrast, high resolution, and no direction limitations compared with other imaging techniques (Liu Y. et al., 2018).

**FIGURE 1:**
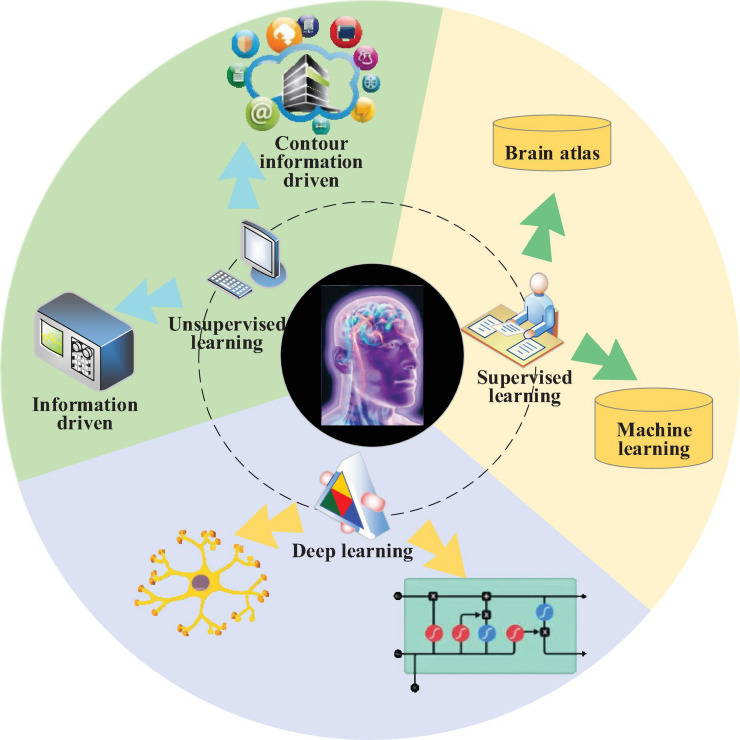
Schematic diagram of brain image segmentation fusion diagnosis.

Regarding brain image processing, excellent brain image segmentation results are of great significance for further target feature extraction and target recognition and analysis. Brain image segmentation differs from ordinary image segmentation in image complexity and diversity (Wang et al., 2018). Brain image segmentation is a crucial branch of medical image segmentation. Key difficulties are as follows: (1) the gray scale distribution between different tissues is not uniform, and the distribution of different tissues overlaps with each other. (2) Brain tissues have complicated structures. Generally, brain MRI images include the brain cortex, gray matter, white matter, cerebrospinal fluid, and other tissues; each has a complicated shape and structure. (3) Boundaries of brain tumors are blurry, and the density distribution is uneven. Thus, in the analysis of brain image processing, the brain image and structure in real space can be mapped to virtual space by digital twins. After the virtual brain image feature extraction and image fusion processing, the treatment scheme for patients with brain diseases can be formulated, and the treatment purpose for patients can be achieved finally. Present brain image segmentation approaches include supervised learning, unsupervised learning, and DL, as illustrated in Figure 1.

To improve the effect of brain image segmentation diagnosis, both unlabeled and labeled data are adopted to put forward an S3VMs regarding the massive volume of unlabeled brain image data based on the above approaches of brain image segmentation diagnosis. S3VMs combine supervised learning and unsupervised learning, which can utilize labeled data and unlabeled data simultaneously to improve the model’s generalization ability (Liu L. et al., 2018; Zavrak and İskefiyeli, 2020). Combining semi-supervised learning and Support Vector Machines (SVMs) based on labeled samples obtained by time-domain simulation and the unlabeled samples from the actual system to assess brain image data can extract actual data distribution comprehensively and improve the weak generalization ability of present supervised feature extraction and assessment models, in an effort to enhance model’s adaptability to the actual system. This idea can solve the shortcomings of the current supervised ML feature extraction models and the starting point of the present work.

### Performance of S3VMs in Brain Image Feature Extraction

Semi-supervised learning combines supervised learning and unsupervised learning. It utilizes both labeled data and unlabeled data for learning and mine the data distribution hidden in unlabeled samples such as brain images, thereby guiding the training of the classifier and improving its performance.

A graph-based semi-supervised learning algorithm is adopted to express the relationships between the entire training data during brain image feature extraction. Graph nodes represent training samples, including labeled samples and unlabeled samples. There are edges between nodes; these edges are given weights to represent the similarity between data nodes and can be obtained using various distance metrics. Overall, the greater the weight, the greater the similarity. No connection between nodes means zero similarity between them. Similar vertices are given the same category labels as much as possible so that the graph distribution is as smooth as possible.

**FIGURE 2:**
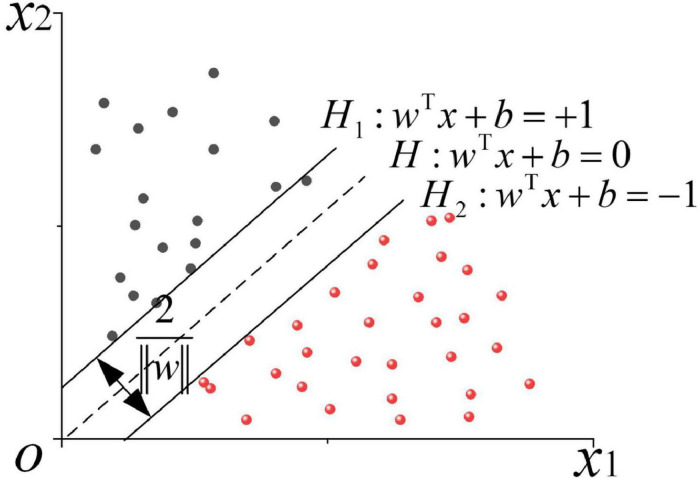
Schematic diagram of the linearly separable optimal classification hyperplane.

The mathematical model of standard SVM can be formalized as a convex quadratic programming problem; then, a linear classification machine can be obtained through hard interval maximization learning (Zhang et al., 2020). If the training data are non-linear and inseparable, the kernel function will be utilized to map the input space to the feature space, and the linear SVM is implicitly learned in the high-dimensional feature space by maximizing the soft interval. The linearly separable optimal classification hyperplane is demonstrated in Figure 2.

As shown in Figure 2, a linearly separable sample set *T* = {(*x*_1_,*y*_1_),⋯,(*x*_*n*_,*y*_*n*_)} is given, where (*x*_*i*_,*y*_*i*_) refers to the sample point, *x*_*i*_ ∈ ^*Rn*^, and *y*_*i*_ ∈ { + 1,−1},*i* = 1,2,⋯,*n*. The red and the black circles represent two types of training samples, respectively. At this point, there is an infinite number of interfaces that can correctly separate the two types of samples. Hence, an optimal classification hyperplane is required, which should not only ensure the minimum empirical risk and correctly divide the two types of samples but also minimize the actual risk and meet the maximum classification interval. The core idea of linear separable SVM employs the maximum classification interval to find the optimal hyperplane, at which the solution is unique. In Figure 2, H denotes the optimal classification hyperplane, H1 and H2 are the planes containing the closest sample points (support vectors) to the classification hyperplane. H1 and H2 are parallel to H, and the distance between them is called the classification interval. The classification hyperplane function of linear separable SVM is:

(1) w⋅x+b=0

In Eq. 1, *w* represents the normal vector of the hyperplane, and *b* denotes the bias. The following conditions must be met to classify samples accurately and ensure the maximum interval simultaneously:

(2) $$y_{i}\,\left(w\cdot x+b\right)\geq 1$$

The classification interval can be obtained as 2/1I y} II. Solving the optimal hyperplane of the training sample set T can be transformed into a problem described by the following equation:

(3) $$\begin{array}{c} \min_{a,b}\frac{{||w||}^{2}}{2}\\ \begin{array}{cc} s.t. & y_{i}\,\left(w\cdot x+b\right)\geq 1 \end{array},i=1,2,\cdots ,n \end{array}$$

Equation (3) is a quadratic programming problem, which can be solved as a dual Lagrangian problem. Introducing the Lagrangian function can obtain:

(4) $$\begin{array}{c} L\,\left(w,b,\alpha \right)=\frac{{||w||}^{2}}{2}-\sum_{i=1}^{n}\alpha_{i}\,\left[y_{i}\,\left(w\cdot x+b\right)-1\right]\\ \begin{array}{cc} s.t. & \alpha_{i}\geq 1 \end{array},i=1,2,\cdots ,n \end{array}$$

In Eq. 4, α_*i*_ refers to the Lagrangian multiplier. The partial derivative of the Lagrangian function can be obtained. According to the extreme conditions:

(5) $$\begin{array}{c} \frac{\partial L}{\partial b}=0\to \sum_{i=1}^{n}y_{i}\,\alpha_{i}=0\\ \frac{\partial L}{\partial w}=0\to w=\sum_{i=1}^{n}y_{i}\,\alpha_{i}\,x_{i} \end{array}$$

Substituting equation 5 into 4 can get the dual problem about the original problem:

(6) $$\begin{array}{cc} \min_{\alpha } & \frac{1}{2}\,\sum_{i=1}^{n}\sum_{j=1}^{n}y_{i}\,y_{j}\,\alpha_{i}\,\alpha_{j}\,\left(x_{i}\cdot x_{j}\right)-\sum_{i=1}^{n}\alpha_{i}\\ s.t. & \sum_{i=1}^{n}y_{i}\,\alpha_{i}=0,\alpha_{i}\,y_{i}\geq 0,i=1,2,\cdots ,n \end{array}$$

Solving Eq. 6 can get the optimal solution $\alpha^{*}={\left(\alpha_{1}^{*},\cdots ,\alpha_{n}^{*}\right)}^{\text{T}}$. Calculating *w*^∗^ and *b*^∗^ can obtain the optimal classification function:

(7) $$f\,\left(x\right)=s\,g\,n\,\left[\sum_{i=1}^{n}\alpha_{i}^{*}\,y_{i}\,\left(x_{i}\cdot x\right)+b^{*}\right]$$

According to Eq. 7, the optimal classification plane of SVM only depends on the sample point (*x*_*i*_,*y*_*i*_) corresponding to $\alpha_{i}^{*}>0$ in the training data. SVM’s optimal classification plane only depends on corresponding sample points in the training data, and these samples just fall on the interval boundary. Usually, the sample point *x*_*i*_ ∈ *R*^*n*^ at $\alpha_{i}^{*}>0$ is called the support vector. The graph feature extraction of S3VMs is analyzed further.

A training set including labeled data and unlabeled data $\left\{{\left(x_{i},y_{i}\right)}_{i=1}^{l},{\left(x_{j}\right)}_{j=l+1}^{l+u}\right\}$ is given. Suppose that *g* = (*V*,*E*) refers to a graph, *V* represents the node set, and *E* describes the edge set. The weight matrix *W* of *g* is symmetric, which can be described as:

(8) $$W_{i\,j}=\left\{ \begin{array}{cc} w\,\left(e\right)=w_{i\,j}=0 & \begin{array}{cc} i\,f & e=\left(x_{i},x_{j}\right)\in E \end{array}\\ 0 & o\,t\,h\,e\,r\,w\,i\,s\,e \end{array}\right.$$

The weight of the edge *e* = (*x*_*i*_,*x*_*j*_)*w*(*e*) = *w*_*ij*_ = *w*_*ji*_ signifies the similarity between nodes *x*_*i*_,*x*_*j*_, which can be calculated using K Nearest Neighbor (KNN) graph. Each vertex in the KNN graph is only connected to its k nearest neighbors under a particular distance (such as Euclidean distance). Notably, a vertex *x*_*i*_ that is the *k* nearest neighbor of *x*_*j*_ does not necessarily indicate that *x*_*j*_ is the k nearest neighbor of *x*_*i*_. While constructing the KNN graph, if *x*_*i*_ and *x*_*j*_ are k-nearest neighbors of each other, *x*_*i*_ and *x*_*j*_ will be connected by an edge, and the corresponding weights will be calculated using the Radial Basis Function (RBF) kernel:

(9) $$W_{i\,j}=\exp\left(-\frac{{||x_{i}-x_{j}||}^{2}}{2\,\sigma^{2}}\right)$$

In Eq. 9, σ refers to the kernel function that controls the weight reduction speed. When *x*_*i*_ = *x*_*j*_, the weight *w*_*ij*_ = 1; when ∣∣*x*_*i*_−*x*_*j*_∣∣ approaches infinite, the weight *w*_*ij*_ = 0.

**FIGURE 3:**
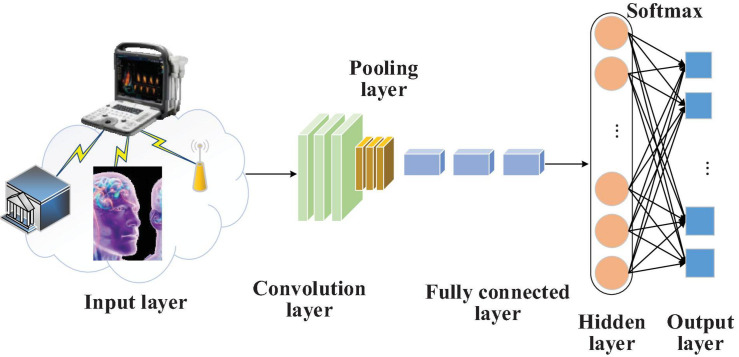
Flowchart of extracting and classifying the data features in brain image DTs based on CNN.

To extract and forecast the obtained brain image features, CNN is introduced into the brain image feature extraction and diagnosis system. The proposed model can minimize the preprocessing workload and directly extract the most expressive features from the original data input without manual feature designation (Ogino et al., 2021). Figure 3 illustrates how CNN extracts and classifies the features of the graph data in brain image DTs.

Convolutional Neural Network pooling layer performs down-sampling operations on the input feature maps in length and width dimensions. It can reduce the model parameters by down-sampling the input feature data. According to the number of parameters, the data complexity of different brain images can be reduced, diminishing the over-fitting degree and the probability of local minimum. Moreover, the pooling layer can make the model more robust to translation and distortion in the image.

Data in CNN go through multiple convolutional layers and pooling layers; then, they are connected via one or more fully connected layers. All neurons in the current layer are connected to those in the last layer. Usually, this layer depends on two 1D network layers. Local information of the convolutional layer or the pooling layer is grouped together. The activation function used by all neurons is often the Rectified Linear Unit (ReLU).

AlexNet (Tanveer et al., 2019), a deep CNN model, is selected considering its multiple network layers and stronger learning ability to reduce the calculation amount and enhance CNN’s generalization performance. Furthermore, the functional layer of AlexNet’s convolutional layer is improved. The operation of “local normalization before pooling” is advanced to “pooling before local normalization.” This improvement brings two benefits. First, the generalization ability of AlexNet can be enhanced while the over-fitting can be weakened, which greatly shortens the training time. Second, overlapping pooling before local normalization can reserve more data and weaken redundant information during pooling and accelerate the convergence rate of the brain image DTs diagnosis and forecasting model during training, highlighting its superiority over other pooling approaches.

### Brain Image Fusion DTs Diagnosis and Forecasting Model Based on S3VMs and Improved AlexNet

**FIGURE 4:**
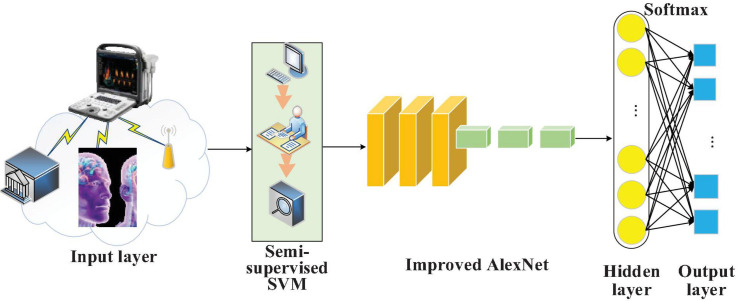
Schematic diagram of the brain image DTs diagnosis and forecasting model based on S3VMs and improved AlexNet.

Digital Twins (Sun et al., 2020) can forecast and analyze data of the physical space in the corresponding virtual space regarding the smart city development in physical spaces. To extract the data features from brain images, AlexNet, a deep CNN model, is selected considering its multiple network layers and stronger learning ability. Furthermore, the functional layer of AlexNet’s convolutional layer is improved. A brain image DTs diagnosis and forecasting model is designed based on S3VMs and improved AlexNet while considering the model’s security performance, as demonstrated in Figure 4.

In this diagnosis and forecasting model, different penalty coefficients are provided considering the different contribution degrees of the labeled and unlabeled data samples in the brain images to the hinge loss. The objective function can be written as the following equation:

(10) $$\begin{array}{cc} \min_{w,b,s,y_{u}} & \frac{1}{2}\,w^{T}\,w+\lambda_{1}\,\sum_{i=1}^{n}u_{i\,i}\,\left(1-\left(w^{T}\,x^{i}+b\right)\,y_{i}\right)\\ & +\lambda_{2}\,t\,r\,\left(w^{T}\,X\,L_{s}\,X^{T}\,w\right)+\lambda_{3}\,{||s||}_{2}^{2}+\lambda_{4}\,t\,r\,\left(F^{T}\,L_{s}\,F\right)\\ s.t. & \forall i,s_{i}\,1\geq 0,F\in R^{n\times c},F^{T}\,F=I \end{array}$$

**FIGURE 5:**
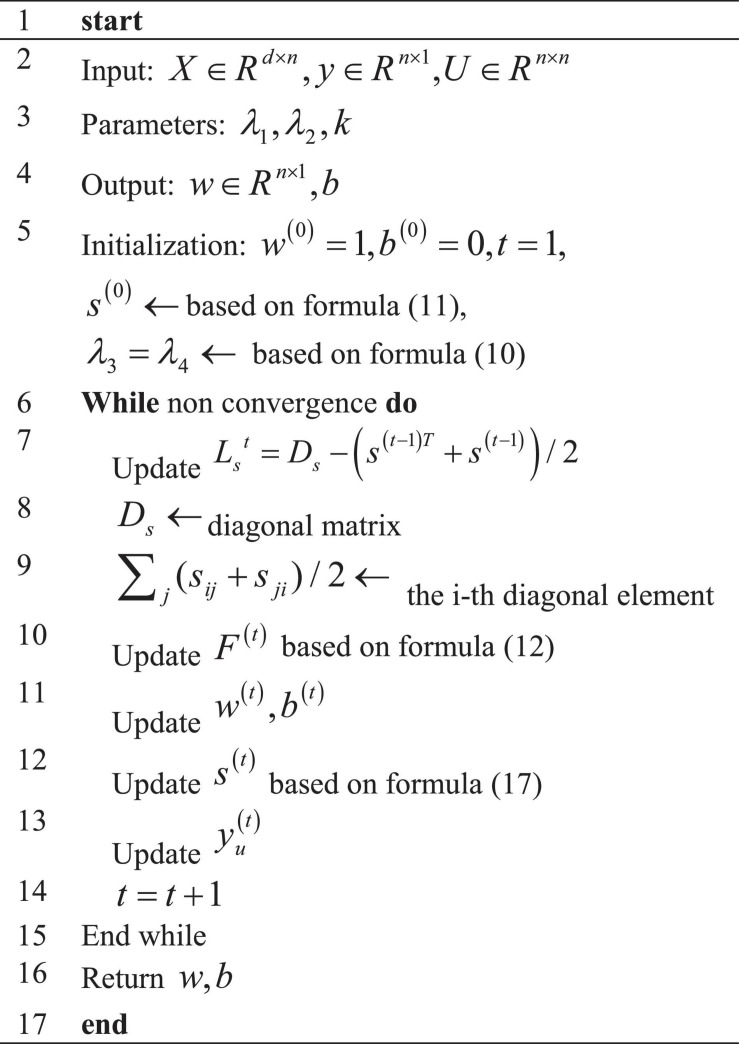
Algorithm flow of objective function’s iterative optimization.

In Eq. 9, *s* signifies a similarity matrix between samples, $L_{s}=D_{s}-\frac{s^{T}+s}{2}$, and *D*_*s*_ ∈ *R*^*n*×*n*^ refers to a diagonal element, the diagonal matrix of $\sum_{j}\left(s_{i\,j}+s_{j\,i}\right)/2\leftarrow y_{u}^{\left(t\right)}\,t=t+1$. The above objective function can be solved by iterative optimization. The optimization process is described in Figure 5.

In this algorithm flow, λ_3_ = λ_4_ and *s* are initialized:

(11) $$\lambda_{3}=\lambda_{4}=\frac{1}{n}\,\sum_{i=2}^{n}\left(\frac{k}{2}\,d_{i,k+1}^{x}-\frac{1}{2}\,\sum_{j=1}^{k}d_{i\,j}^{x}\right)$$

(12) $$s_{i\,j}=\left\{ \begin{array}{cc} 0, & x^{j}\notin N_{k}\,\left(x^{i}\right)\\ \frac{d_{i,k+1}^{x}-d_{i\,j}^{x}}{k\,d_{i,k+1}^{x}-\sum_{j=1}^{k}d_{i\,j}^{x}}, & x^{j}\in N_{k}\,\left(x^{i}\right) \end{array}\right.$$

Here, $d_{i\,j}^{x}={||x^{i}-x^{j}||}_{2}^{2}$. The smallest top *k* $d_{i\,j}^{x}$ are found by sorting in increasing order, and *N*_*k*_(*x*^*i*^) refers to the *k* samples nearest to *x*^*i*^. *F*^(*t*)^ is updated as the following equation:

(13) $$\begin{array}{cc} \min_{F} & t\,r\,\left(F^{T}\,L_{s}\,F\right)\\ & s.t.F\in R^{n\times c},F^{T}\,F=I \end{array}$$

Eigenvectors corresponding to the smallest *c* eigenvalues of the Laplacian matrix *L*_*s*_ can form *F*.

Solving *s*^(*t*)^ only needs to solve Eq. 13:

(14) $$\begin{array}{cc} \min_{s_{i}\,1=1,s_{i}\geq 0} & \sum_{i=1}^{n}\left(\lambda_{2}\,{||w^{T}\,x^{i}-w^{T}\,x^{j}||}_{2}^{2}\,s_{i\,j}+2\,\lambda_{3}\,s_{i\,j}^{2}\right)\\ & +\sum_{i=1}^{n}\lambda_{4}\,{||f_{i}-f_{j}||}_{2}^{2}\,s_{i\,j} \end{array}$$

Features of brain images are extracted. The *t*-th feature map $y_{t}^{l}\,\left(i,j\right)$ of the *l*-th convolutional layer is sampled using overlapping pooling:

(15) $$\begin{array}{c} a_{t}^{l}\left(i,j\right)=\max\left\{y_{t}^{l}\left(i,j\right),i_{s}\leq i\leq i_{s}+w_{c}-1,\right\}\\ j_{s}\leq j\leq j_{s}+w_{c}-1 \end{array}$$

In Eq. 14, *s* is the pooling movement step size, *w*_*c*_ refers to the width of the pooling area, and *w*_*c*_ > *s*.

A local normalization layer is added after the first and second pooling layers of AlexNet to standardize the feature map $c_{t}^{l}\,\left(i,j\right)$:

(16) $$c_{t}^{l}\,\left(i,j\right)=a_{t}^{l}\,\left(i,j\right)/{\left(k+\alpha \,\sum_{\max\left(0,t-m/2\right)}^{\min\left(N-1,t+m/2\right)}{\left(a_{t}^{l}\,\left(i,j\right)\right)}^{2}\right)}^{\beta }$$

In Eq. 15, *k*,α,β,*m* are all hyperparameters valuing 2, 0.78, 10^–4^, and 7, respectively, and *N* is the total number of convolution kernels in the *l*-th convolutional layer. To prevent “gradient dispersion” (Shen et al., 2019), the activation function takes Rectified Linear Unit (ReLU) to activate the convolution output $S_{t}^{l}\,\left(i,j\right)$:

(17) $$y_{t}^{l}\,\left(i,j\right)=f\,\left(S_{t}^{l}\,\left(i,j\right)\right)=\max\left\{0,S_{t}^{l}\,\left(i,j\right)\right\}$$

In Eq. 16, *f*( ) represents ReLU. To prevent over-fitting in the fully connected layer, the dropout parameter is set to 0.5. All the feature maps when *l* values 5 in Eq. 12 are reconstructed into a high-dimensional single-layered neuron structure *C*^5^; thus, the input $Z_{i}^{6}$ of the *i*-th neuron in the sixth fully connected layer is:

(18) $$Z_{i}^{6}=W_{i}^{6}\,C^{5}+b_{i}^{6}$$

In Eq. 17, $W_{i}^{6}$ and $b_{i}^{6}$ are the weight and bias of the *i*-th neuron in the sixth fully connected layer, respectively.

While improving the generalization ability, the neurons *C*^*l*^ of the sixth and seventh fully connected layers are discarded and output, $r_{j}^{l}∼b\,e\,r\,n\,o\,u\,l\,l\,i\,\left(d\,p\right),{\tilde{C}}^{l}=r^{l}\,C^{l}$; in this regard, the *i*-th neuron’s input in the seventh and eighth fully connected layers $Z_{i}^{l+1}$ is $W_{i}^{l+1}\,{\tilde{C}}^{l}+b_{i}^{l+1}$, where the *i*-th neuron’s input in the sixth and seventh fully connected layers $C_{i}^{l}$ is $f\,\left(Z_{i}^{l}\right)$, namely $\max\left\{0,Z_{i}^{l}\right\}$. Finally, the input *q*^*i*^ of the *i*-th neuron in the eighth fully connected layer can be obtained:

(19) $$q^{i}=s\,o\,f\,t\,\max\left(Z_{i}^{8}\right)=\frac{e^{Z_{i}^{8}}}{\sum_{j=1}^{12}e^{Z_{i}^{8}}}$$

The cross-entropy loss function suitable for classification is taken as the model’s error function, and the equation is:

(20) $$L\,o\,s\,s=\sum_{i=1}^{K}y_{i}\cdot \log\left(p_{i}\right)$$

(21) $$p_{i}=\frac{\exp\left({\tilde{y}}_{i}\right)}{\sum_{i=1}^{K}\exp\left({\tilde{y}}_{j}\right)}$$

In Eqs. 19 and 20, *K* denotes the number of categories, *y*_*i*_ describes the true category distribution of the sample, ${\tilde{y}}_{i}$ describes the network output, and *p*_*i*_ represents the classification result after the SoftMax classifier. SoftMax’s input is an *N*-dimensional real number vector, denoted as *x*. Its equation is:

(22) $$\xi \,{\left(x\right)}_{i}=\frac{e^{x_{i}}}{\sum_{n=1}^{N}e^{x_{i}}},i=1,2,\ldots ,N$$

Essentially, SoftMax can map an *N*-dimensional arbitrary real number vector to an *N*-dimensional vector whose values all fall in the range of (0,1), thereby normalizing the vector. To reduce the computational complexity, the output data volume is reduced to 2^8^ through μ companding conversion, that is, μ = 255, thereby improving the model’s forecasting efficiency.

(23) $$f\,\left(x_{t}\right)=s\,i\,g\,n\,\left(x_{t}\right)\,\frac{\ln\left(1+\mu \,\left|x_{t}\right|\right)}{\ln\left(1+\mu \right)},\left|x_{t}\right|<1$$

The proposed model is trained through learning rate updating using the polynomial decay approach (Poly) (Zhao et al., 2020). The equation is:

(24) $$i\,n\,i\,t\,\_\,l\,r\times {\left(1-\frac{e\,p\,o\,c\,h}{\max\_\,e\,p\,o\,c\,h}\right)}^{p\,o\,w\,e\,r}$$

In Eq. 23, the initial learning rate *init*_*lr* is 0.0005 (or 5e^–4^), and power is set to 0.9.

The Weighted Cross-Entropy (WCE) is accepted as a cost function to optimize model training. Suppose that *z*_*k*_(*x*,θ) describes the unnormalized logarithmic probability of pixel *x* in the *k*-th category under the given network parameter θ. In that case, the SoftMax function *p*_*k*_(*x*,θ) is defined as:

(25) $$p_{k}\,\left(x,\theta \right)=\frac{\exp\left\{z_{k}\,\left(x,\theta \right)\right\}}{\sum_{k^{\prime }}^{K}\exp\left\{z_{k^{\prime }}\,\left(x,\theta \right)\right\}}$$

In Eq. 24, *K* represents the total number of image categories. During forecasting, once Eq. 24 reaches the maximum, pixel *x* will be labeled as the *k*-th category, namely *k*^∗^ = *arg*⁡*max*⁡{*P*_*k*_(*x*,θ)}. A semantic segmentation task needs to sum the pixel data loss in each input mini-batch. Here, *N* denotes the total number of pixels in the training batch of image data, *y*_*i*_ refers to the real semantic annotation of the pixel *x*_*i*_, and *p*_*k*_(*x*_*i*_,θ) describes the forecasted probability of pixel *x*_*i*_ belonging to the *k*-th semantic category, that is, the log-normalized probability, abbreviated as *p*_*ik*_. Hence, the training process aims to find the optimal network parameter θ^∗^ by minimizing the WCE loss function ℓ(*x*,θ), denoted as $\theta^{*}=\min_{\theta }\ell \,\left(x,\theta \right)$. Training samples with unbalanced categories in brain images usually make the network notice some easily distinguishable categories, resulting in poor recognition on some more difficult samples. In this regard, the Online Hard Example Mining (OHEM) strategy (Ghafoori et al., 2018) is adopted to optimize the network training process. The improved loss function is:

$$\ell \,\left(x,\theta \right)=-\frac{1}{\sum_{i=1}^{N}\sum_{k=1}^{K}\delta \left(y_{i}=k,p_{i\,k}<\eta \right)}$$

(26) $$\sum_{i=1}^{N}\sum_{k=1}^{K}\delta \left(y_{i}=k,p_{i\,k}<\eta \right)\log p_{i\,k}$$

In Eq. 25, η ∈ (0,1] refers to the predefined threshold, and δ(⋅) describes the symbolic function, which will take 1 if the condition is met and 0 otherwise. The weighted loss function for brain image fusion is defined as:

(27) $$\ell \,\left(x,\theta \right)=-\sum_{i=1}^{N}\sum_{k=1}^{K}w_{i\,k}\,q_{i\,k}\,\log p_{i\,k}$$

In Eq. 26, *q*_*ik*_ = *q*(*y*_*i*_ = *k*|*x*_*i*_) denotes the true label distribution of the *k*-th category of the pixel *x*_*i*_, *w*_*ik*_ refers to the weighting coefficient. The following strategy is employed during training:

(28) $$w_{i\,k}=\frac{1}{\ln\left(c+p_{i\,k}\right)}$$

In Eq. 27, *c* is an additional hyperparameter, set to 1.10 based on experience during simulation experiments.

### Simulation Experiment

In the present work, MATLAB is adopted for simulation to validate the performance of the proposed model. Brain image data in experiments come from MRI records of the Brain Tumor Department of a Hospital. It contains 20 groups of clinical data, and each group of data includes four kinds of multi sequence MRI images: T1, T2, Tlc, and FLAIR. The selected image data are processed by registration, skull peeling, contrast enhancement and other operations. Each case of MRI image contains the standard segmentation results given by artificial experts. Data collected are divided into a training dataset and a test dataset in 7:3; the ratio of each data type in the two datasets shall be consistent. Hyperparameters of AlexNet are set as follows: 120 iterations, 2,000 seconds of simulation, and 128 Batch Size. Some state-of-art models are included for performance comparison, including LSTM (Nguyen et al., 2021), CNN, RNN (Minerva et al., 2021), AlexNet, and MLP (Dong et al., 2019). Experimental environment configuration includes software and hardware. As for software, the operating system is Linux 64bit, the Python version is 3.6.1, and the development platform is PyCharm. As for hardware, the Central Processing Unit (CPU) is Intel Core i7-7700@4.2 GHz 8 Cores, the internal memory is Kingston DDR4 2400 MHz 16G, and the Graphics Processing Unit (GPU) is NVIDIA GeForce 1060 8G.

The similarity between the experimental segmentation result and the expert segmentation result can measure the model’s segmentation quality (Dai et al., 2020). Common similarity metrics are the Jaccard coefficient and Dice Similarity Coefficient (DSC). DSC indicates the similarity degree between the segmentation result of the model and the label labeled by the expert. The higher the DSC, the higher the segmentation similarity. Positive Predictive Value (PPV) represents the proportion of correct brain tumor points in the experimental segmentation to the tumor points in the segmentation result. Sensitivity describes the proportion of the correct tumor points in the experimental segmentation to the true value of the tumor points. The above indicators are adopted to assess the proposed brain image segmentation model. The Jaccard coefficient, DSC, PPV, and Sensitivity are defined as follows:

(29) $$J\,a\,c\,c\,a\,r\,d\,\left(T,P\right)=\frac{\left|T\,\wedge \,P\right|}{\left|T\vee P\right|}$$

(30) $$D\,S\,C\,\left(T,P\right)=\frac{2\,\left|T\,\wedge \,P\right|}{\left|T\right|+\left|P\right|}$$

(31) $$P\,P\,V=\frac{\left|T\,\wedge \,P\right|}{P}$$

(32) $$S\,e\,n\,s\,i\,t\,i\,v\,i\,t\,y=\frac{\left|T\,\wedge \,P\right|}{T}$$

In Eqs. 28–31), *T* represents the expert segmentation result, and *P* denotes the segmentation result of the proposed model based on S3VMs and the improved AlexNet.

## Results and Discussion

### Forecasting Performance Analysis

**FIGURE 6:**
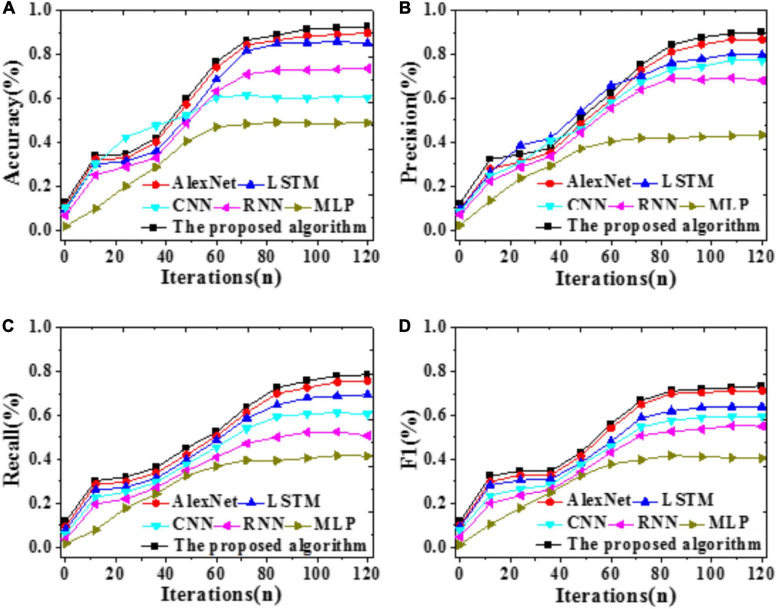
Recognition accuracy of different models with iterations **(A)** Accuracy; **(B)** Precision; **(C)** Recall; **(D)** F1.

**FIGURE 7:**
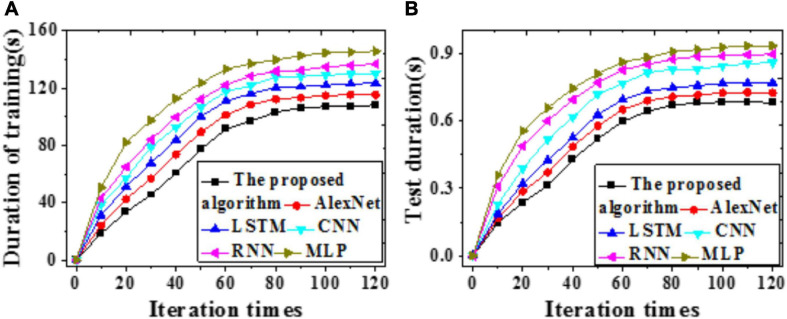
Time duration required by different models **(A)** Training duration; **(B)** Test duration.

**FIGURE 8:**
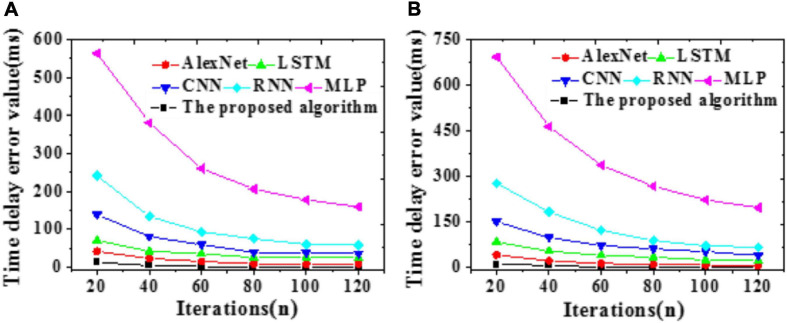
Time delay errors of different models **(A)** Training set; **(B)** Test set.

To explore the forecasting performance of the proposed model, LSTM, CNN, RNN, AlexNet, and MLP are included for comparison. Values of Accuracy, Precision, Recall, and F1 of these models are compared through simulation experiments, and the results are illustrated in Figure 6. Furthermore, time durations required for training and test and errors are compared, as in Figures 7, 8.

As shown in Figure 6, the proposed model can provide a recognition accuracy of 92.52%, at least an improvement of 2.76% than other models. The Precision, Recall, and F1 of the proposed model are the highest, at least 3.35% higher than other models. Hence, the proposed brain image DTs diagnosis and forecasting model based on S3VMs and improved AlexNet can provide excellent recognition and forecasting accuracy.

According to Figures 7, 8, the time required by all models first increases then stabilizes with iterations. Compared with LSTM, AlexNet, CNN, RNN, and MLP, the proposed model requires remarkably less time for training and test. A possible reason is that the improved AlexNet provides enhanced generalization ability while accelerating the convergence velocity of the model training process. Therefore, the proposed model can achieve higher forecasting accuracy more quickly.

In both the training and test sets, errors reduce gradually with iterations. MLP provides the longest time delay, 564.15 ms and 693.06 ms, respectively. In contrast, the proposed model provides a time delay approaching zero, the smallest among all tested models. Comparisons of RMSE and MAE values are summarized in Tables 1, 2 below:

**TABLE 1:** RMSE (%) changes of each model with iterations.

	**Iterations**		
	**1.00**	**60.00**	**120.00**
The proposed model	4.85	5.09	4.78
AlexNet	5.61	5.79	5.31
LSTM	6.43	6.11	6.01
CNN	7.28	6.51	6.45
RNN	7.99	6.87	7.04
MLP	8.42	7.33	7.48

**TABLE 2:** MAE (%) changes of each model with iterations.

	**Iterations**		
	**1**	**60**	**120**
The proposed model	5.67	5.45	5.66
AlexNet	6.59	6.98	6.91
LSTM	8.92	8.26	8.44
CNN	9.93	9.11	8.92
RNN	10.53	9.59	9.48
MLP	11.22	10.19	10.01

According to Tables 1, 2, the RMSE and MAE of the proposed model are 4.91 and 5.59%, respectively, significantly lower than other models. Hence, it can provide stronger robustness and better accuracy for brain image diagnosis and forecasting.

### Comparing Assessment Indicators of Brain Image Segmentation and Fusion

**FIGURE 9:**
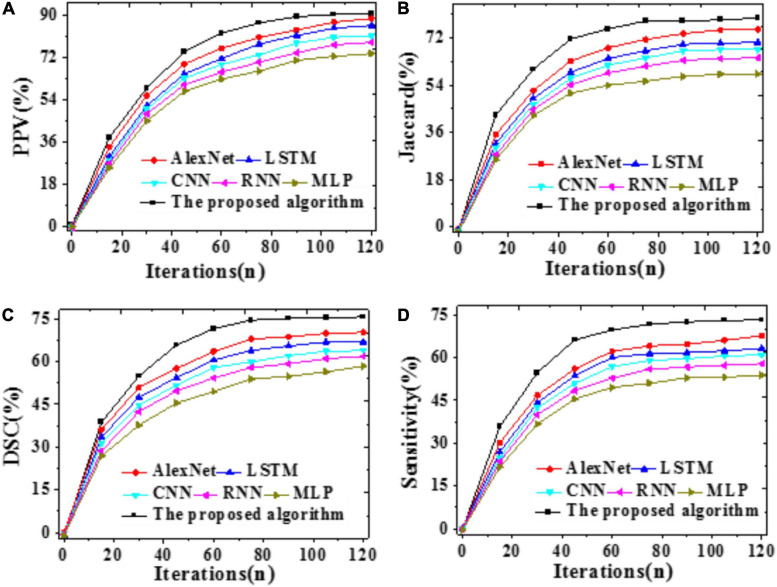
Brain image assessment indicators of different models with iterations **(A)** The Jaccard coefficient; **(B)** DSC; **(C)** PPV; **(D)** Sensitivity.

The Jaccard coefficients, DSCs, PPVs, and Sensitivity of the proposed model, LSTM, CNN, RNN, AlexNet, and MLP are compared. The results are demonstrated in Figure 9.

The higher the indicator value, the more accurate the segmentation result. As shown in Figure 9, the proposed model can provide a 79.55% Jaccard coefficient, a 90.43% PPV, a 73.09% Sensitivity, and a 75.58% DSC, remarkably better than other models. Hence, the proposed model outperforms other state-of-art models for MRI brain tumor image segmentation.

### Acceleration Efficiency Analysis

**FIGURE 10:**
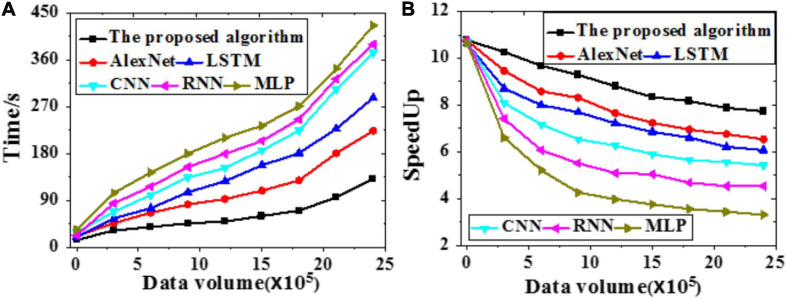
Time required and speedup indicator of different models under different data volumes **(A)** Time required; **(B)** Speedup indicator.

The acceleration efficiency of the proposed model is analyzed through simulation experiments. The time required and speedup indicator of different models under different data volumes are presented in Figure 10.

The proposed model is less sensitive to brain image data growth than other models. Hence, it is suitable to process massive data. Simultaneously, the larger the data volume, the higher the speedup indicator. To sum up, the proposed model can provide better data recognition and segmentation results than other models.

A diagnosis and prediction model of brain image fusion digital twins based on semi supervised SVM and improved AlexNet is proposed to improve the accuracy of traditional CNN segmentation of MRI brain tumor images. The model mainly includes the preprocessing of brain image data based on semi supervised SVM. Then, the improved AlexNet network algorithm is used to extract and analyze the brain image features. Finally, through the comparative test and verification of simulation experiments, the model algorithm constructed can acquire more accurate segmentation results and prediction accuracy, and can effectively solve a series of problems in the process of MRI brain tumor image fusion, such as under-segmentation and over-segmentation, and meet the needs of clinical pathological diagnosis.

## Conclusion

Brain tumor images have complicated edge structures, artifacts, offset fields, and other defects that affect image segmentation. Extracting features from multi-sequence MRI brain tumor images becomes particularly vital. Both unlabeled and labeled data are utilized to construct the S3VMs regarding the massive number of unlabeled brain image data. Afterward, AlexNet is improved, and a brain image fusion DTs diagnosis and forecasting model is built based on S3VMs and improved AlexNet. Simulation experiments are performed to analyze the proposed model’s performance. Results demonstrate a 92.52% feature recognition and extraction accuracy, a 79.55% Jaccard coefficient, a 90.43% PPV, a 73.09% Sensitivity, and a 75.58% DSC of the proposed model, providing an experimental basis for brain image feature recognition and digital diagnosis. There are some weaknesses in the present work. The constructed model does not grade brain tumors. In the future, the texture feature, shape feature, position information, and volume of the multi-sequence MRI image will be extracted based on brain tumor segmentation results before being classified by ML and DL algorithms, achieving the key steps of precise brain tumor treatment and truly meeting the clinical needs, which will provide remarkable significance for the subsequent clinical diagnosis and treatment of brain tumors.

## Data Availability Statement

The original contributions presented in the study are included in the article/supplementary material, further inquiries can be directed to the corresponding author.

## Author Contributions

ZW was responsible for the writing and implementation of the full text. YD was responsible for the implementation of the experiment. ZY was responsible for the collection of data and the writing of related research. HL was responsible for the implementation and data analysis of the experiment. ZL was responsible for the design of the experiment and the inspection of the results.

## Conflict of Interest

YD was employed by Qingdao Haily Measuring Technologies Co., Ltd., The remaining authors declare that the research was conducted in the absence of any commercial or financial relationships that could be construed as a potential conflict of interest.
